# A draft framework for measuring progress towards the development of a national health information infrastructure

**DOI:** 10.1186/1472-6947-5-14

**Published:** 2005-06-13

**Authors:** Dean F Sittig, Richard N Shiffman, Kevin Leonard, Charles Friedman, Barbara Rudolph, George Hripcsak, Laura L Adams, Lawrence C Kleinman, Rainu Kaushal

**Affiliations:** 1 Department of Medical Informatics, Northwest Permanente, P.C., Portland, OR USA; 2 Center for Medical Informatics, Yale University School of Medicine, New Haven, CT USA; 3 Department of Health Policy, Management & Evaluation, University of Toronto, Toronto, Ontario Canada; 4 National Library of Medicine, Rockville, MD USA; 5 The Leapfrog Group, Washington, DC USA; 6 Department of Biomedical Informatics, Columbia University, New York, NY USA; 7 Rhode Island Quality Institute, Providence, RI USA; 8 Center for Research Evaluation and Planning, Nemours Foundation, Newark, DE USA; 9 Division of General Internal Medicine, Brigham and Women's Hospital, Boston, MA USA; 10 Department of Public Health, Cornell Medical School, NY, NY USA

## Abstract

**Background:**

American public policy makers recently established the goal of providing the majority of Americans with electronic health records by 2014. This will require a National Health Information Infrastructure (NHII) that is far more complete than the one that is currently in its formative stage of development. We describe a conceptual framework to help measure progress toward that goal.

**Discussion:**

The NHII comprises a set of clusters, such as Regional Health Information Organizations (RHIOs), which, in turn, are composed of smaller clusters and nodes such as private physician practices, individual hospitals, and large academic medical centers. We assess progress in terms of the availability and use of information and communications technology and the resulting effectiveness of these implementations. These three attributes can be studied in a phased approach because the system must be available before it can be used, and it must be used to have an effect. As the NHII expands, it can become a tool for evaluating itself.

**Summary:**

The NHII has the potential to transform health care in America – improving health care quality, reducing health care costs, preventing medical errors, improving administrative efficiencies, reducing paperwork, and increasing access to affordable health care. While the President has set an ambitious goal of assuring that most Americans have electronic health records within the next 10 years, a significant question remains "How will we know if we are making progress toward that goal?" Using the definitions for "nodes" and "clusters" developed in this article along with the resulting measurement framework, we believe that we can begin a discussion that will enable us to define and then begin making the kinds of measurements necessary to answer this important question.

## Background

In the United States of America, public policy makers recently established the goal of having electronic health records (EHRs) for the majority of Americans by 2014 [1]. This article presents recommendations regarding specific aspects of a conceptual and measurement framework that will help us to measure progress toward that goal. It represents a starting point for what will hopefully be a wide-ranging discussion of exactly how we should measure progress toward the achievement of a functional National Health Information Infrastructure (NHII) [Note: Other terms have also been used to describe this rather nebulous concept including National Health Information Network (NHIN), National Health Information Infrastructure (NHII), Regional Health Information Organization (RHIO), and Local Health Information Infrastructure (LHII), we will use NHII to refer to this concept. Such an NHII would allow all patients, healthcare providers, and those interested in population health to have access to comprehensive electronic health records. This article resulted from a discussion at "The Secretarial Summit on Health Information Technology Launching the National Health Information Infrastructure 2004: Cornerstones for Electronic Healthcare" held in Washington, D.C., July 20–23, 2004

### What is the NHII?

The National Health Information Infrastructure (NHII) is:

• An initiative set forth to improve the effectiveness, efficiency and overall quality of health and health care in the United States

• A comprehensive knowledge-based network of interoperable systems of clinical, public health, and personal health information that would improve decision-making by making health information available when and where it is needed.

• The set of technologies, standards, applications, systems, values, and laws that support all facets of individual health, health care, and public health [2].

Measuring the progress in creation, deployment and adoption of health information management and communications technology in support of the healthcare delivery process across the nation will be difficult [3]. As we move from the individual patient's health record to those contained in the entire practice of that patient's primary care physician and the hospital at which that physician practices, to the entire health system in which that hospital participates, to the entire region in which that health system exists, to the entire nation, we will likely be forced to accept less precision in our measurements.

Following an overview of a conceptual model for the NHII, we present a draft measurement framework that would allow us to begin measuring progress towards the successful creation of a fully functional NHII. We will then briefly describe how we might also try to develop a qualitative estimate of the current state of the art regarding various information exchange standards, current and impending legislation, and the "values" of potential users of these systems.

## Discussion

### A conceptual model of the NHII

"Human endeavor is caught in an eternal tension between the effectiveness of small groups acting independently and the need to mesh with the wider community" [4]. The NHII can be thought of as a collection of healthcare delivery providers that share patient-level information electronically. More specifically, we conceptualize the NHII as a cluster of nodes. We define a node as a physical healthcare environment with the requisite health information management technology to collect, store, display and transmit patient-identifiable, structured, clinical data in an electronic format. Therefore, a sole practitioner in private practice using a simple, electronic health record (EHR) system who has access to the Internet could function as a node. On the other hand, we would also consider a large, academic medical center's inpatient facility as a single node, as well.

To create a functional network infrastructure, individual nodes must be connected in a way that permits sharing of information. Connections rely on the application of agreed upon conventions, or standards for describing clinical and administrative information (i.e., controlled vocabularies, standard identifiers) and for transmitting that information electronically (i.e., message exchange standards, e.g., HL-7 and X.12). The 1996 Health Insurance Portability and Accountability Act and subsequent regulations defined several standardized approaches [5]. Recent research on the growth and behavior of networks suggests we should anticipate significant increases in the capability of these networks as the number of connections grows [6].

**Figure 1:**
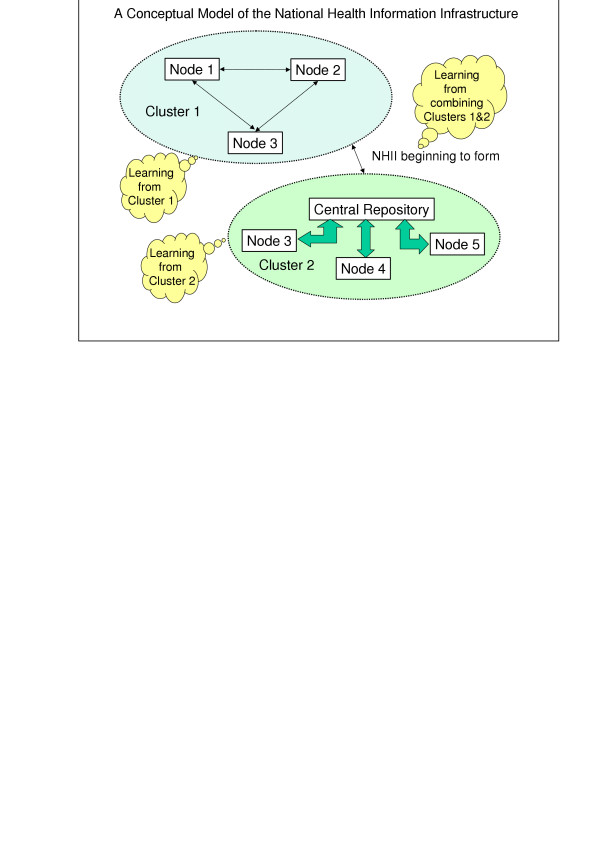
**A conceptual model of the National Health Information Infrastructure**. A conceptual model of the National Health Information Infrastructure that illustrates different types of NHII clusters (i.e., one with peer to peer connections the other with a central repository). Once these clusters begin linking up that is the beginning of the NHII.

We define a cluster as two or more nodes that have a) an existing written data sharing agreement and b) sent (or received) patient-identifiable information to (or from) any other node in the cluster – either directly or through an intermediary which in this case serves as a hub through which others share information. [Note: The process is more than a single claims submission transaction, users must also be able to retrieve, or at least view information from others.] A node may belong to one or more clusters (see Figure 1 for an illustration showing how nodes and clusters can be related). Those aspects of a cluster that contribute to their persistence also help to define clusters. For example, a cluster may be created and maintained by one or more of the following attributes: statutory, or legal, agreements, geographic proximity, or financial ownership. Using this definition, several existing Local Health Information Infrastructures (LHIIs) or Regional Health Information Organizations (RHIOs) would be considered clusters (e.g., the Indianapolis Network for Patient Care (INPC), the Santa Barbara County Care Data Exchange (CDE) or MA-SHARE (Massachusetts Simplifying Healthcare Among Regional Entities [7]).

As we go forward, we anticipate that groups of clusters will form; therefore we add the additional proviso that a cluster can consist of a cluster of clusters. Such a model encapsulates the U.S. Federal government's current articulated plan for achieving a National Health Information Infrastructure (NHII) through the creation of Local or Regional Health Information Organizations (RHIOs) [8].

### Key users (stakeholders) of the NHII

The National Committee on Vital and Health Statistics (NCVHS) defined key dimensions of the NHII functionality "by what they encompass, whom they serve, how they are used, and who has primary responsibility for content and control" [9]. These dimensions helped them identify three major groups of users of patient-identifiable health information: patients or consumers, healthcare providers (both individual clinicians and organizations) and communities (or population/public health). Therefore, we believe that we must make measurements with respect to each of these three groups of users.

### Using the conceptual model to create a measurement framework

**Figure 2:**
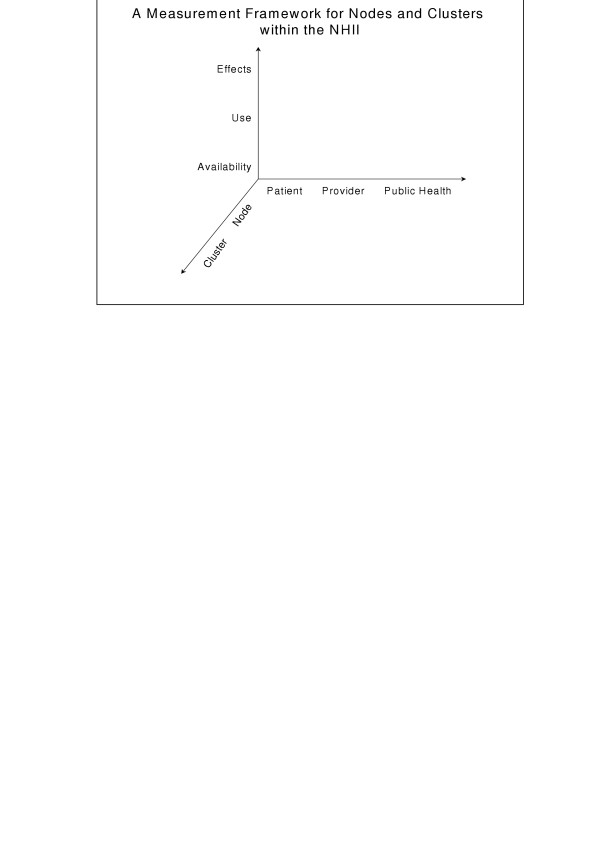
**A measurement framework for nodes and clusters with the NHII**. An illustration of a measurement framework for nodes and clusters within the NHII showing the 3 axes along which measurements should be made: health information management technology availability, use, and effectiveness; NHII level, for example node and cluster; key users of the NHII, namely, patients, clinicians, and those involved in population health activities (e.g., public health departments).

Now that we have a conceptual model for the NHII, we can begin developing a measurement framework that will help us evaluate the nation's progress toward achieving a functional NHII. Borrowing several concepts from conventional quality measurement efforts, we must be able to measure aspects of the structure, process, and outcomes that make up and result from the NHII [10]. These concepts translate into measurements of health information management technology availability, use, and effectiveness at both the nodal and cluster level. In addition, all of these measurements need to be made from the viewpoints of the key users of the NHII, namely, patients, clinicians, and those involved in population health activities (e.g., public health departments). Figure 2 helps illustrate this concept. As in any large-scale measurement and evaluation effort, designing and validating the measures will be one of the most important and difficult challenges to overcome.

### Broadening the conceptual framework to help us better understand the field

The application of information technology to health care is still in its youth. Although much has been learned over the years, there are still many lessons to be learned about how and when a computer-based intervention is most likely to be successful. The scope of harms and benefits to be anticipated when information technology is implemented has not been well catalogued. The development of such understanding represents a key aspect of the formative evaluation of the move towards NHII. One further aspect of our measurement framework borrows from both the case study and the quality improvement frameworks. The accumulation of data from RHIOs and specific initiatives ought to enhance our understanding both of how and when to implement a specific type of intervention in a particular environment, and to improve the nature of the technological innovations themselves.

### Phased approach to making measurements

In addition to the conceptual model of the system and identification of the key system users, we believe that we should use an iterative, phased approach, that will allow us to begin making measurements of the NHII, while we continue learning "how best to make these measurements". This iterative approach will also allow us to move forward at varying rates in different regions of the country. This is based on our firm belief that before one can expect to demonstrate improvements in any of the outcome measures associated with the NHII, we must first demonstrate that the key system users are actually using the system. Similarly, we believe that before we can expect to be able to measure any system use, we must be able to demonstrate that the requisite systems are in place and available to our key users. Therefore, we propose a three-phase iterative approach to beginning the measurements: Phase I will consist of the measurements required to demonstrate "Availability" of the systems; Phase II will consist of the measurements required to demonstrate "Use" of the systems; and Phase III will consist of the measurements required to demonstrate the effect of these systems on various outcome measures that are often associated with health information technology (HIT) use.

### Phase I – Systems availability

HIT availability can be defined as the existence of, and access to, the requisite technology to collect, store, display and transmit patient-identifiable, structured, clinical data in electronic formats. Therefore, we must be able to identify whether healthcare institutions and their providers have access to various health information technology components. Potential measurements that we could make in this phase include:

• What is the coverage, or percentage, of patients in a region who have their health data available in an electronic format to qualified personnel? As our measurement techniques become more sophisticated, we also hope to be able to measure, or at least estimate, the "completeness" of each patient's health record, although at the present time the definition of a "complete" electronic medical record is still not precisely defined. [Note: On September 1, 2004, the American Health Information Management Association, Healthcare Information and Management Systems Society, and The National Alliance for Health Information Technology announced the formation of a Certification Commission for Healthcare Information Technology. Their charge is to create an efficient, impartial and trusted mechanism for certifying ambulatory electronic health records and other healthcare information technology (IT) products. It is possible that an EHR "completeness" measure could formulated based on their recommendations.]

• Use U.S. census data for a geographic region covered to estimate denominator.

• Use number of unique patient ID's accessible in the system(s) as the numerator.

• Goal: identify 3–5 levels of coverage.

• Number or percentage of clinicians with an RHIO login?

• Use number of unique clinicians with a log-in as numerator.

• Use state licensure records as an estimate of total clinicians in region eligible for logins.

• Goal to identify 3–5 levels.

• Number or percentage of health care organizations in a geographic region with a signed data exchange agreement with the RHIO in place.

• Use total number of healthcare organizations in community as denominator.

• Count the numbers of these LHIIs or RHIOs nationally – perhaps we could even go back a few years and make estimates for 2001–2003

### Phase II – Systems use

HIT use can be defined as actual hands-on use of these HIT systems by patients, providers, and those involved in population health. At the nodal level this equates to actual use of various HIT applications such as clinical results review or provider order entry. At the cluster level, HIT use can be measured by the number of clinicians who routinely use the system to enter and review patient-level data. Example measurements we might be able to make here include:

• Patients in a region whose data was accessed by someone other than the originator of the data.

• Clinicians who actually logged-in to the system

• Healthcare institutions that submitted data to the RHIO

### Phase III – Effect measurement

The effects of health-related information technology on health and health care represent a vital metric for the NHII. The value of the infrastructure ultimately must be evaluated perhaps using the six quality attributes defined in [11] (i.e., Safety, Timeliness, Efficiency, Effectiveness, Equitability, Patient-centeredness) as measurement axes. Although benefits and costs of HIT have been measured in limited settings, measurements of effect on the scale envisioned for a national infrastructure have never been made. We believe, however, that measurements of the impact of NHII on health outcomes are beyond the scope of our current charge and may distract us from the critical measurements of systems availability and use that must be performed first.

### Paying for the RHIOs and the NHII

Clearly, HIT requires significant financial resources to create and maintain it. Therefore, we must be able to at least estimate how much each node or cluster has spent to create and maintain their systems and services and their source of financing. Using these financial estimates, we can then begin to compare different RHIO models based on their return on investment.

### When the NHII is up and running

Once we have considerable (e.g., > 25%) penetration of the NHII, then we can begin using electronic, randomly determined, sampling methods of various aspects of the NHII systems to generate objective measures of IT availability, use and effectiveness. For example, we could send queries for a statistically significant number of patients' data (at least one patient in this group should have data from each hospital selected) to randomly selected hospitals and measure both the number and quality of responses received. The number of responses would tell us how many hospitals were able to at least respond to queries of this type, which is essential. The quality of the responses, that is the sensitivity and specificity of the patient matches and the amount and nature of the data returned would tell us how effectively, these institutions had implemented the functionality required to implement such a system.

• Randomly select a statistically significant number of patients and send electronic requests for information to a randomly selected set of healthcare institutions, pharmacies, or labs and count the number of replies. This would provide an estimate of the number of institutions that were capable of working in this system.

• Use the National Provider Identification (NPID) database to estimate number of duplicates as a measure of how well this database is being managed.

### Additional measurement features

In addition to the measurements associated with elements of the conceptual model described earlier, we also believe that our measurements of NHII progress should include qualitative reviews of the current state of the art with regard to the legislation that is in place, or impending. Likewise, we believe that similar qualitative studies should be conducted on the state of clinical and administrative information exchange standards and on the "values" of potential users of these systems. While these qualitative estimates of progress will not be as easy to interpret, they provide at least a glimpse of the progress that the nation is making in these critical arenas.

Examples of the types of topics these qualitative reviews might address include:

• Qualitative assessment of the legal climate in each state to support NHII

• Patient privacy protections

• Legal restrictions on sending/receiving various data types

• Electronic signatures

• Prescription transmission to pharmacies

• Legal restrictions on sending laboratory results to patients

• Requirements to submit data in electronic format to local, state, federal payers

• Availability of unique provider ID at federal level

Likewise in assessing the values of key system users one might delve into:

• Qualitative assessment of the perceived value of using HIT for patient care

• Incentives to adoption

• Number of insurance companies reimbursing physicians for use of e-visits

### How will we define these measurements?

We recommend that the United States department of Health and Human Services (HHS) convene and co-sponsor an impartial, public-private partnership group, such as an Institute of Medicine (IOM) committee, to create clear and consistent definitions of the components of the NHII (e.g., RHIO, EHR, CPOE) as a basis for the further refinement of specific metrics. This group should be lead by a recognized leader in the field of clinical information systems measurement and evaluation. The remainder of the group should include experts who have expertise in, and can represent and advocate for various measurement perspectives. Key stakeholders from various governmental agencies, healthcare delivery systems, and patient advocacy groups should advise this group.

The group should be charged with defining a set of metrics and developing a methodology to test their reliability and validity. They should release a base set of metrics as soon as they are defined and agreed upon. They should also work to define additional test metrics that are released, but not required to be made for the initial baseline estimates.

### Who will make these measurements?

A public-private partnership should be charged with further development of these measurement systems, making the measurements, and reporting the results of these measures on a yearly basis.

For example, a nascent group referred to as the Improve-IT Institute  has been formed by two of the authors (DFS and KL). Briefly, ImproveIT is an international coalition of institutions and individuals focused on measuring the progress in adoption and utilization of clinical information technology.

### How can or should these measurements be made?

Making measurements of such a multi-faceted, multi-functional set of disparate systems and services will be difficult. Until we have considerable penetration in all aspects of these systems (i.e., inpatient, outpatient, data interchange standards, and unique patient ID mechanisms) measurements will need to be estimated from survey or site visit data.

### What should be done first?

We should begin creating a multi-level inventory of NHII components including functionality and interoperability. This inventory should also include estimates of the population covered. We should also create a website documenting existing RHIOs and a means for sharing best practices.

## Summary

The NHII has the potential to transform health care in America – improving health care quality, reducing health care costs, preventing medical errors, improving administrative efficiencies, reducing paperwork, and increasing access to affordable health care. While the President has set an ambitious goal of assuring that most Americans have electronic health records within the next 10 years, a significant question remains "How will we know if we are making progress toward that goal?" Using the definitions for "nodes" and "clusters" developed in this article along with the resulting measurement framework, we believe that we can begin a discussion that will enable us to define and then begin making the kinds of measurements necessary to answer this important question.

## List of abbreviations

NHII – National Health Information Infrastructure

RHIOs – Regional Health Information Organizations

EHRs – Electronic Health Records

NHIN – National Health Information Network

LHII – Local Health Information Infrastructure

INPC – Indianapolis Network for Patient Care

CDE – Care Data Exchange

MA-SHARE – Massachusetts Simplifying Healthcare Among Regional Entities

NCVHS – National Committee on Vital and Health Statistics

HIT – Health Information Technology

IT – Information Technology

NPID – National Provider Identification

HHS – United States department of Health and Human Services

IOM – Institute of Medicine

CPOE – Computer-based Provider Order Entry

Improve-IT – Indices to Measure Performance Relating Outcomes, Value and Expenditure generated from Information Technology

## Competing interests

The Improve-IT Institute  has been formed, and is owned, by two of the authors (DFS and KL). Their goal is to further develop the ideas outlined in this manuscript and begin making and reporting the results of these measurements on their website. All of the other authors declare that they have no competing interests.

## Authors' contributions

DFS, RNS, KL, CF, BR, GH, LLA, and LCK participated in several conference calls during which the concept for the paper and main ideas were conceived. DFS drafted the manuscript. DFS, RNS, KL, CF, BR, GH, and RK participated in the review and discussion of the "Metrics" topics described in this article at The Secretarial Summit on Health Information Technology Launching the National Health Information Infrastructure 2004: Cornerstones for Electronic Healthcare" held in Washington, D.C., July 20–23, 2004. DFS, RNS, and RK developed the final recommendations. All authors read and approved the final manuscript.

## Pre-publication history

The pre-publication history for this paper can be accessed here:


